# Sensors and Sensory Processing for Airborne Vibrations in Silk Moths and Honeybees

**DOI:** 10.3390/s130709344

**Published:** 2013-07-19

**Authors:** Hiroyuki Ai

**Affiliations:** Department of Earth System Science, Faculty of Science, Fukuoka University, 8-19-1 Nanakuma, Jonan-ku, Fukuoka 814-0180, Japan; E-Mail: ai@fukuoka-u.ac.jp; Tel.: +81-92-871-6631; Fax: +81-92-865-6030

**Keywords:** vibration, wingbeat, bristle, proprioceptors, *Bombyx*, Johnston's organ, antenna, waggle dance, honeybee, brain

## Abstract

Insects use airborne vibrations caused by their own movements to control their behaviors and produce airborne vibrations to communicate with conspecific mates. In this review, I use two examples to introduce how insects use airborne vibrations to accurately control behavior or for communication. The first example is vibration-sensitive sensilla along the wing margin that stabilize wingbeat frequency. There are two specialized sensors along the wing margin for detecting the airborne vibration caused by wingbeats. The response properties of these sensors suggest that each sensor plays a different role in the control of wingbeats. The second example is Johnston's organ that contributes to regulating flying speed and perceiving vector information about food sources to hive-mates. There are parallel vibration processing pathways in the central nervous system related with these behaviors, flight and communication. Both examples indicate that the frequency of airborne vibration are filtered on the sensory level and that on the central nervous system level, the extracted vibration signals are integrated with other sensory signals for executing quick adaptive motor response.

## 1. Introduction

Insects control their behavior by using various kinds of sensory organs to detect environmental information. Among others, they use mechanosensory organs, which exist around the body for triggering escape behavior, controlling walking and flight. The wings of flying insects contain several mechanosensory organs, which all provide feedback on wingbeats during flight. For example, during locust flight, the inputs from stretch receptors on the base of the wings induce excitatory postsynaptic potentials (EPSPs) on the elevator muscle and inhibitory postsynaptic potentials (IPSPs) on the depressor muscle and compensate the frequency and amplitude of wingbeats [1]. Moreover, the tegulae on the base of the hindwings monitor wing movements by detecting distortion of the cuticles caused by the wingbeat for regulating the timings of depressing and elevating wings during the wingbeats [2]. By combining electrophysiological experiments and computational modeling, it has been demonstrated that tegula feedback strength determines the wingbeat frequency such that stronger feedback results in lower frequencies [3]. Similarly, flies synchronize their wingbeat with the input of the halters, their gyroscopic mechanosensory organs, for specific ranges of stimulus amplitude and frequency [4]. In general, rapid mechanosensory feedback can tune cyclic motor patterns on a cycle-by-cycle basis [5]. It has been suggested that also the equally spaced bristles along the wing margin of lepidopterous species (butterfly and moth) are sensory sensilla related to the control of wingbeats. However, until now their mechanical responsiveness remain unknown. Recently, the authors found that these sensory bristles are sensitive to airborne vibrations around the wing margin [6].

Conversely, some insects use airborne vibration caused by wingbeats for communication. The wingbeat frequencies of these insects are species-specific, and in some cases sex-specific, and are a key stimulus in conspecific and sex recognition. For example, male *Aedes aegypti* mosquitoes are very sensitive to airborne vibrations caused by conspecific females (beat frequency 380 Hz) [7]. Moreover, the male mosquito can distinguish immature and mature females based on the frequency of their wingbeats. The fruit fly performs courtship songs by vibration one or both wings [8]. Honeybees receive information about distance to a food source from airborne vibrations caused by wingbeats during the species-specific waggle dance. Honeybees have thus evolved the ability to convert the concrete information “distance to the food source” into the symbol “duration of airborne vibration during the dance” for sharing profitable information in the hive. How do the honeybees decode the distance information from the airborne vibration? The sensor and sensory processing of airborne vibration are critical topics to answer this question.

Johnston's organ (JO), located on the pedicel of antennae of several insect species, is specialized for detecting airborne vibrations. This mechanosensory organ is a stretch receptor (chordotonal organ) for detecting distortion on the base of the flagella [9]. Recently, molecular biological methods have revealed that the majority of JO development, structures, and molecules related to transforming the distortion of the cuticle into electrical signals, are common with those in mammalian cochlear hair cells. For this reason, insects including *Drosophila* have become animal models in auditory processing research [10]. Moreover the neural mechanisms relating to these processes have been clarified. This paper will review how insects detect airborne vibrations and produce adaptive behaviors by looking at two examples: the vibration-sensitive bristles along the wing margin of moth, and Johnston's organ in honeybee antennae. The characteristics of airborne vibration processing in insects will also be discussed.

## 2. Vibration-Sensitive Bristles along the Wing Margin of Silkmoth

Insect flight has attracted the attention of many neuroethologists and has been the subject of numerous behavioral studies because of the unique machinery used for flight and sophisticated orientation behavior. Among them, the sex-pheromone-guided orientation behaviors of moths have been extensively studied using wind tunnels [11-17]. However, there have been few neurophysiological studies of the proprioceptive mechanoreceptors related to moth flight. A stretch receptor, one of proprioceptive mechanoreceptors at the wing hinge of *Manduca sexta*, detects the position of the elevating wing [18-20]. The signals from the stretch receptor are thought to be integrated with descending visual cues, ultimately controlling the wing kinematics and resultant aerodynamic force production during flight [20]. This implies that proprioceptive mechanoreceptors play important roles in regulation of the flight motor pattern. The wings of several lepidopterous species have three kinds of morphologically different sensilla: the sensory bristle, the sensory scale, and the campaniform sensillum [21-23]. Specifically, it was speculated that the arrangement of sensory bristles along the wing margin might be suitable for detecting the wingbeat in male *Pieris rapae*[23]. Although there was no physiological evidence of their function, we found in experiments that the neurons innervating the bristles along the wing margin of male *P. rapae* and *Bombyx mori* were excited only by vibratory wind stimuli, but not by stationary bending of the bristles [6].

### 2.1. The Distribution and Morphologies of Bristles on the Wing Margin

We examined the morphology and physiology of sensory bristles in the moth *B. mori* to clarify the functions and roles of these wing margin bristles, for the following reasons: (1) The experiments in non-flying *B. mori* are easier to perform than in flying *P. rapae*. (2) The arrangement of the wing sensory bristles in male *B. mori* silkworm moths is very similar to that in male *P. rapae*[6] suggesting that the bristles in both insects may have similar functions. (3) The arrangements and motor patterns of *Bombyx* flight muscles are the same as those of the other lepidopterous insects [24,25], *Bombyx* is considered to be a suitable model for studies on insect flight motion. Therefore, it is anticipated that the experimental results may be applicable to both the other moths and butterflies. In this review we introduce the morphological and electrophysiological characteristics of the bristles along wing margin of the male *B. mori* and discuss the roles of the bristle in relation to wingbeat and flight.

**Figure 1.**
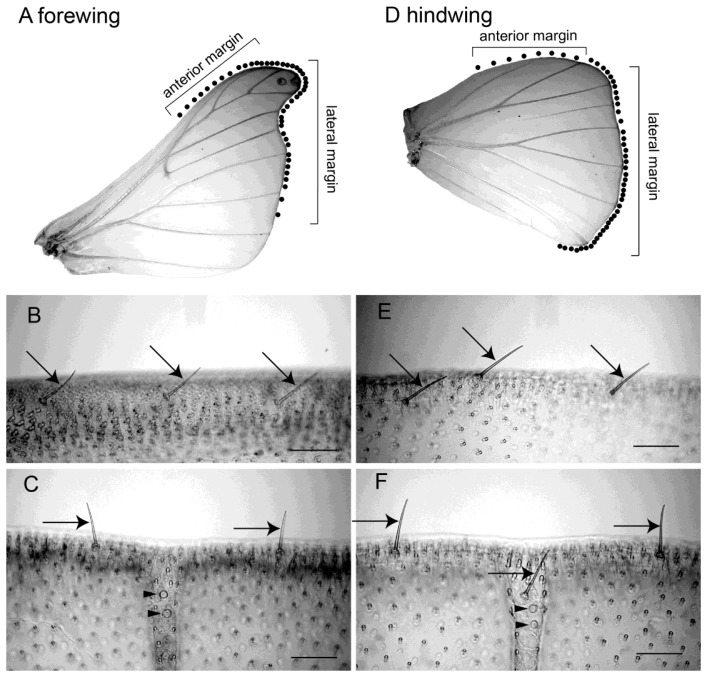
(**A**) Distribution of the bristles along the wing margin on the forewing and (**B**) on the hindwing *of Bombyx mori*. Dots show the positions of the bristles. All of the bristles are located on the ventral side of the wing margin. There are 45 bristles on the forewing and 50 bristles on the hindwing. (**C**) The bristles aligned along the anterior margin of the forewing and (**D**) along the lateral margin of forewing. (**E**) Campaniform sensilla on the lateral ends of the wing veins (arrowheads). Scale bars: 100 μm. Modified from Ai *et al.*[6].

There were ca. 45 bristles on the forewing and ca. 55 bristles on the hindwing of male *B. mori* (Figure 1A,B). The bristles were observed ventrally along the margins of both wings at intervals of 200-800 μm (Figure 1A,B). Results from microscopic observations showed that the bristles along the wing margin were typical mechanosensilla that had the following common characteristics. The bristles was externally composed of a socket and a shaft, which was smooth-surfaced without any cavities or pits. In the socket of the bristles there were single sensory cell for each sensillum, which is also typical of the mechanosensilla of insects [6]. The lengths of the the bristles were estimated to be approximately 100 μm, and the diameters of their socket were approximately 5 μm. The bristles along the wing margin are uniform with respect to the length of the shaft (ca. 100 μm) and in the diameter of socket (ca. 5 μm). For example, the filiform hairs on the cerci have a variable shaft length (30-150 μm) and socket diameter (1.5-9 um) [26]. The response characteristics of the sensory neuron in mechanoreceptive sensilla are closely related to the size of the hair. For example, crickets are able to detect a wide range of intensities, velocities and frequencies of air currents with these sensilla [27]. It is widely considered that the filiform hair is involved in triggering escape behavior through being a mechanosensory bristle that detects fine air-particle movements from an approaching predator. By contrast, the bristles along the wing margin are uniform and only suitable for detecting a narrow range of air current intensities, velocities and frequencies.

Moreover, the bristles on the anterior margin were arranged at ca. 40 degrees against the tangential line of the wing margin Figure 1C and those on the lateral margin at ca. 90 degrees against the tangential line of the wing margin Figure 1D. The fact that the orientation of the bristles is the same depending on the region of the wing suggests that these bristles along the wing margin function as detectors air currents with a limited range of direction. Given the numerous other types of sensilla along the wing margin, such as the sensory scale and campaniform sensillum Figure 1E, it is suggested that wings play a role not only as effectors for flight but also as sensory organs. How then do bristles along the wing margin respond to mechanical stimuli and what role do they play in insect behavior?

### 2.2. Characteristics of Vibration Responsiveness of Bristles along the Wing Margin

Our studies of how sensory neurons in the bristles along the wing margin respond to vibrations have revealed that neurons respond to airborne vibrations but not to constant air current [6]. The sensory neuron discharged spikes in a tonic manner during stimulation with vibratory air currents from a perpendicular direction of the body axis to the bristles along the wing margin. During wingbeat and flight there will be continuous aerodynamic flows around the wings. A study on the aerodynamics of the tobacco hawkmoth *M. sexta* wing during flight revealed that a continuous air flow runs posteriorly across the lateral margin of the wing parallel to the body axis [28]. If the aerodynamics in *M. sexta* also applies to *Bombyx*, the following may be argued. The deflection axes of the bristle along the postero-lateral margin of the wing are almost parallel to the body axis (upwind stream) because the deflection axes of the bristles in the area are almost parallel to the body axis (ca. 0.6 degrees).

**Figure 2.**
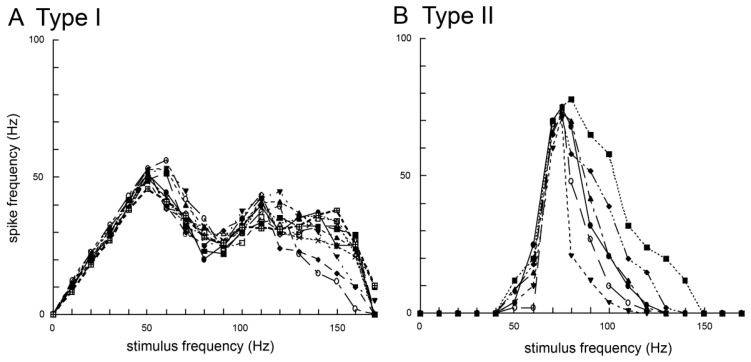
Comparison of the response patterns between neurons in Type I (**A**) and Type II (**B**) bristles along the wing margin of *Bombyx mori*. Each response curve of a different marker was obtained from a different bristle. The vertical axis shows the spike frequencies in the stationary state of the responses, between 2 and 3 seconds from the stimulus onset. The neurons in Type I showed linear correlation between the stimulus frequency and the spike frequency in the range of up to 50 Hz stimulus frequency (n = 11). The neurons in Type II did not fire spikes in the low frequency range but fired one spike for each sinusoidal wave at around 75 Hz stimulus frequency (n = 6). Modified from Ai *et al.*, (2010) [6].

Moreover, experiments on the response properties of bristles along the wing margin when vibrated with different frequencies found at least two types of sensory neurons in the bristles. In these experiments, each bristle along the wing margin was connected to an oscillator and was vibrated with sinusoidal waves in the range 0–200 Hz. The type I bristles showed phasic-tonic response to vibrations and evoked spikes on the same phase of the sinusoidal waves of less than 60 Hz (Figure 2A) [6]. Conversely, the type II bristles evoked spikes on the sinusoidal waves of more than 40 Hz (Figure 2B) [6]. The wingbeat frequency of *B. mori* is ca. 40 Hz [29] and both types of bristles are responsive to airborne vibration caused by the wingbeat. These results show there to be at least two types of vibration-sensitive bristles along the wing margin that can detect wingbeat vibration, and these bristles have unique responsiveness to airborne vibration with restricted frequencies. On the base of the wings there are proprioceptors (*i.e*., stretch receptor) to monitor wing movements during wingbeats. However, the bristles along the wing margin are mechanical sensors monitoring airborne vibrations caused by the wingbeat, which suggests that the bristles along the wing margin have different roles (*i.e.*, the detectors of wingbeat frequency) from that of known proprioceptors such as the stretch receptor (the detector of the wingbeat amplitude).

### 2.3. Roles of Bristles along the Wing Margin in Wingbeat

What roles do bristles along the wing margin play in wingbeat motion? In response to sex pheromones emitted by conspecific females, male *Bombyx* show a stereotyped zigzag walking with constant wingbeat frequency (ca. 40 Hz) and amplitude [30]. If the bristles along the wing margin are rendered motionless by being covered by glue, there is a remarkable decrease in the frequency and amplitude of wingbeats induced by sex pheromone stimuli [31]. These results suggest that the positive feedback inputs from the bristles along the wing margin into the central nervous system (CNS) are necessary for maintaining the constant wingbeat frequency and amplitude. Type I bristles respond to vibrations of less than 60 Hz, suggesting type I has a role in monitoring wingbeat frequency [6]. Type I might be related to the positive feedback system for keeping a constant wingbeat. However, the distance between bristles along the wing margin and the CNS is quite large (ca. 2 cm), and the diameter of sensory axons of the bristles is small, suggesting it will be difficult for the bristles along the wing margin to evoke postsynaptic potential (PSP) on flight motoneurons, per every cycle of the wingbeats as not like the stretch receptors on the base of the wings. The bristles along the wing margin might accelerate the cyclic rhythm of the wingbeats by modulating the central pattern generator (CPG) of wingbeats. Type II bristles respond to vibrations of more than 40 Hz. Because this frequency (40 Hz) corresponds almost to that of the *Bombyx* wingbeat [30], type II bristles is regarded as a kind of sensor to detect the upper limit of wingbeats and could be related to the negative feedback system for preventing excessive beat frequency. These two types of bristles along the wing margin might have the roles of “accelerator” and “brake”, which contribute to the simple regulation system in keeping stable wingbeat motions peripherally without complicated processing in the CNS [6].

## 3. Vibratory Communication of Honeybees

### 3.1. Production of Airborne Vibrations

**Figure 3.**
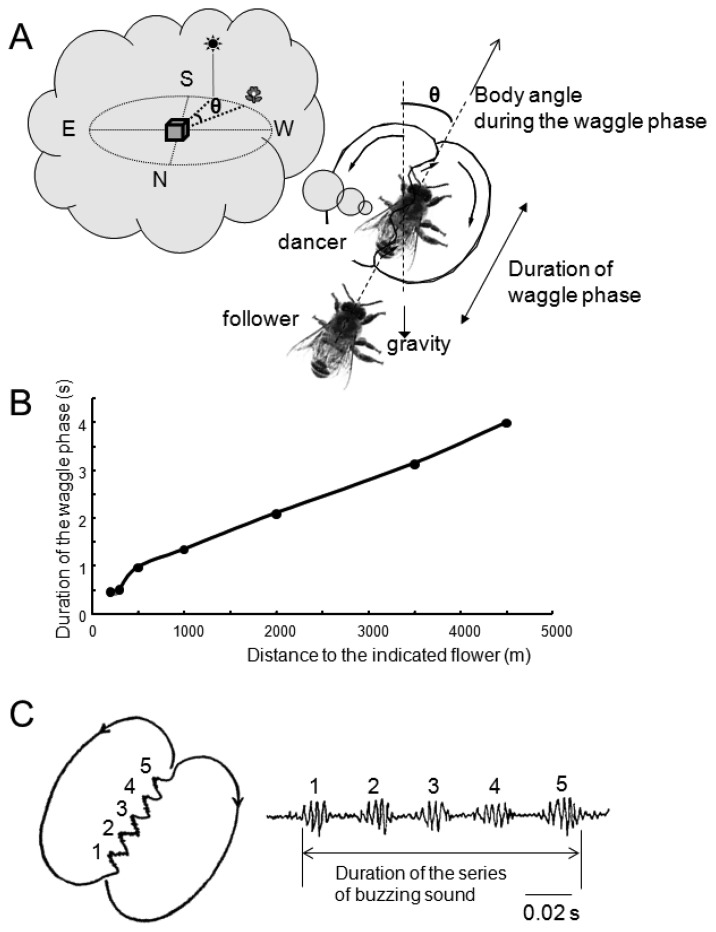
Waggle dance communication of honeybees. (**A**) The forager (dancer) coming back from a foraging trip transfers vector information to hive-mates (followers) by the figure-of-eight dance shown in the image. In this case, the comb stands vertically. In this example, the follower deciphers vector information that the indicated flower is in the direction of θ ° to the west based on the direction of the sun. (**B**) The figure-of-eight dance consists of a straight-line run and return run. This dance is also called a waggle dance because the dancing bee waggles its abdomen from side to side and produces the pulsed thoracic vibration on the straight-line run. (**C**) Frequency spectrum of the pulsed thoracic vibration indicating the main frequency (MF = 0 dB). B and C were adapted with permission from Hrncir *et al.* [34].

The waggle dance of honeybees consists of a straight run with waggle and return run (Figure 3A,B). Von Frisch discovered that foragers sent vector information by waggle dance to their hive-mates, and that the direction of the indicated flower could be encoded in the body orientation during the waggle run and the distance to the flower in the duration of the waggle phase [32]. Moreover, von Frisch demonstrated that the total duration of a series of brief pulses caused by wingbeats during the waggle run was correlated with the distance toward the indicated flower.

Subsequent research found that airborne vibrations are created around the waggle dancer [33]. The dancer performs not only the waggle movements but also wingbeats during the waggle run (Figure 3B) [33]. The frequency of wingbeats (thoracic vibration) is ca. 265 Hz (Figure 3C), and the frequency of waggle movements is 12-15 Hz [34]. These motions create air-jets behind the tail of the dancer. The speed of the air-jets is highest closest to the abdomen and decreases with distance (30 cm/s close to the abdomen, 4 cm/s 5 cm away from the abdomen) [33]. During the waggle dance, followers will align their body with the dancer [35,36], suggesting that bees acquire distance information by detecting the airborne vibrations and air-jets created by the dancer close to her abdomen.

### 3.2. Roles of the Antenna and Johnston's Organ in Vibration Detection

**Figure 4.**
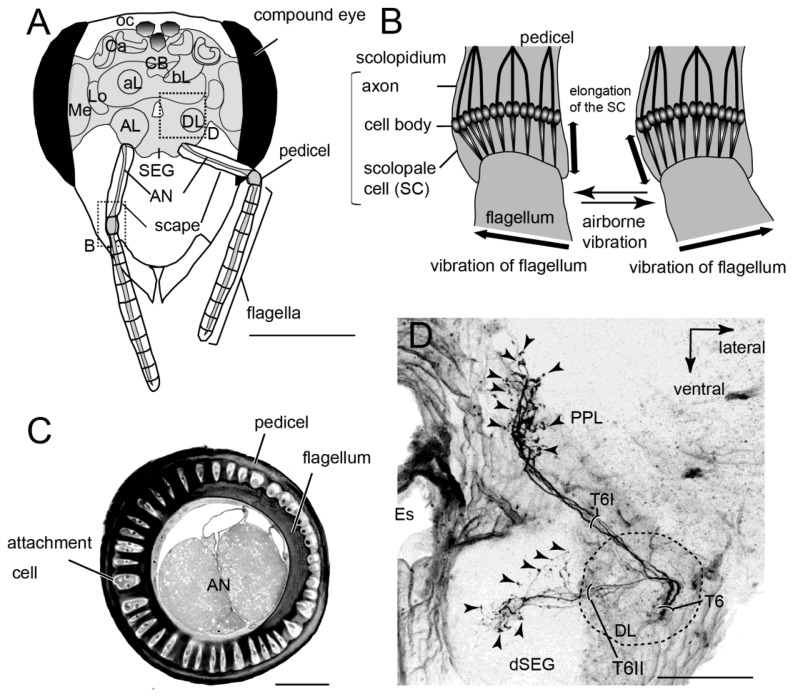
(**A**) Schematic drawing of antennae and brain/subesophageal ganglion (brain-SEG complex) in the honeybee head capsule viewed frontally. The antenna is composed of three parts: the scape, pedicel and flagellum. Johnston's organ (JO) is in the pedicel. The left hemisphere shows the main neuropiles on the anterior plane of the brain through the antennal lobe (AL) and the right hemisphere shows those on the middle plane of the brain through the dorsal lobe and dorsal subesophageal ganglion (DL-dSEG). Dotted box indicate areas of the images shown in (B). AN, antennal nerve; aL, alpha lobe of mushroom body; bL, beta lobe of mushroom body; Ca, Calyx of mushroom body; CB, central body; LO, lobula; Me, medulla, Oc, ocelli. (**B**) Schematic drawing of structures in the JO. The JO is composed of about 240 scolopidia in which each scolopidium has a few JO sensory cells. Airborne movements cause the deflections of the flagellum that stretch the scolopales in the opposite side against the deflections. (**C**) 1.5 μm transverse section of the antenna between the scape and pedicel. The attachment cells extending from the scolopales composing of JO connect with the segments membrane equally around the flagella. AN, antennal nerve. (**D**) Central projection of the JO afferents. These sensory afferents bifurcate into two sub-branches T6I and T6II. The sensory afferents in T6I project into the medial PPL and those in T6II arborize and terminate in the DL and dorsal subesophageal ganglion (dSEG). These are blebby terminals in both areas (indicated by arrowheads). Es, esophagus; PPL, posterior protocerebral lobe; T6, tract 6. Scale bar = 1 mm (A), 50 μm (C), 100 μm (D). Modified from Ai *et al.* [38].

The neural mechanism of honeybee vibration reception has been examined extensively by neuroanatomical and electrophysiological studies. The organ that detects vibrations in honeybees is Johnston's organ (JO), which is located in the antennae. The JO is not directly activated by air currents, but by the resonance of flagellar vibrations caused by air-particle movements. In most insects the 3rd segment (flagellum) is longer than the 2nd segment (pedicel; Figure 4A). In honeybees the pedicel functions as a pivot and a slight displacement at the tip of the flagella evokes distortions at the base of the flagella (Figure 4B). The JO is composed of hundreds of scolopale cells inside the pedicel that detect distortion of the segment between the pedicel and flagella. For example, the displacement threshold for inducing an electrical signal on the sensory cell is 0.7 nm at the tip of the flagella in mosquitoes and 20 nm in honeybees [37]. Because there are approximately 240 scolopales attached around the base of the flagella through attachment cells in the pedicel (Figure 4C), JO could detect airborne vibrations from all directions around the central axis of the antenna.

One of the critical roles of the JO is to detect sound created by the wingbeats of conspecific females, and so the resonance frequency of the flagella is often designed to be equal to that of the wingbeat frequency of conspecifics. The mechanical sensitivities of the antennal flagellum are specifically high in response to low but not high intensity stimuli of 265–350 Hz frequencies, which correspond with the frequency of wingbeats during the waggle run [37]. Even if the amplitude of the 265 Hz vibratory stimuli increases over a certain value, the flagella do not follow the vibration stimuli. This characteristic is almost the same as that of deceased honeybees, suggesting that this is not a mechanism possessed in living cells but rather a mechanical property of the antenna [37]. Tsujiuchi *et al.* speculated that this characteristic is to prevent sensory neurons of JO from being excessively excited [37]. In the waggle dance, because follower bees communicate only when in close proximity to dancers, detection of a slight distortion of the flagella is enough for transfer of information to occur.

### 3.3. Central Projection of Sensory Afferents of JO

Many interneurons in the honeybee brain have been identified morphologically and physiologically. This information has given us knowledge about the functional roles of neuropiles. Therefore it is now possible to speculate on what kinds of processing the sensory signals could be related to. Recently, the authors determined the central projection of sensory afferents of JO in honeybees [38].

Mechanosensory neurons, including JO on the antennae, send axons into the brain-dSEG complex (Figure 4D). There are two pathways of sensory afferents of JO: one is the pathway projecting into the posterior protocerebral lobe (PPL) through T6I, and the other is that projecting into the dorsal lobe-dorsal subesophageal ganglion neuromere (DL-dSEG) through T6II. The PPL is considered to be the opto-motor reflex center, which has many terminal arborizations of visual interneurons and also the dendrites of the motion-sensitive descending neurons. JO functions as the velocity detector during flight, and it is suggested that honeybees regulate flying speed by coordinating velocity detected by JO with visual movements during flight [39]. As one of the terminal regions of the JO afferents, PPL might thus be related to flight.

DL-dSEG has been considered as the center of integration of the antennal mechano- (including vibration) and gustatory-sensing [40]. Trophallactic contacts are often observed before and after waggle dance communication. This behavior is considered to be an olfactory communication for informing hive-mates about the scent and sweetness of nectar. Hive-mates are recruited according to the information acquired by both waggle dance (vector information) and trophallaxis (olfactory information). Dance-following bees detect the direction toward the indicated flower by the dancer's body angle (Figure 3A). A recent study found that the sensory afferents of neck hairs (NHs) detecting the body angle on the vertical comb send axons to the dSEG [41], which is also a central projection area of JO [38]. Moreover, Ai and Hagio [42] demonstrated somatotopic organization within the dSEG of the central projections of the mechanosensory neurons of the NHs. The terminals of the NH afferents in dSEG are in close apposition to those of JO afferents. These results suggest that DL-dSEG might be a critical region for decoding vector information about the indicated flower as communicated in the waggle dance.

### 3.4. Processing of Vibrations in the Primary Center of JO

The authors have conducted morphological and physiological studies of several interneurons related to vibration processing in the primary center of JO [43–45]. In this review, we introduce three types of vibration-sensitive interneurons that have characteristic response patterns.

#### 3.4.1. DL-Int-1

**Figure 5.**
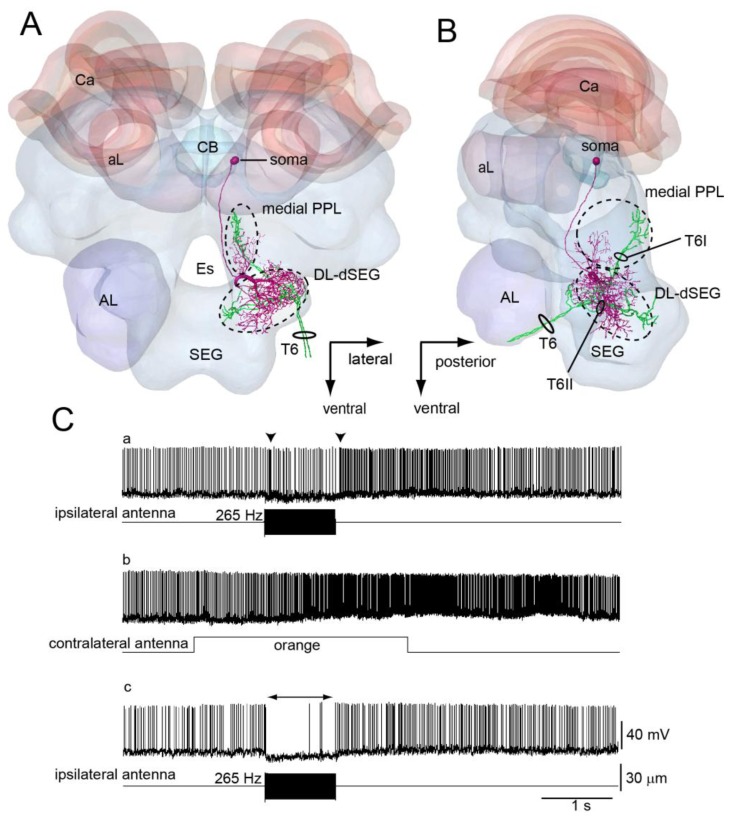
(**A** and **B**): Spatial relationship etween a DL-Int-1 (magenta) and Johnston's organ (JO) afferents (green) (A, frontal view; B, lateral view). The intracellularly marked DL-Int-1 and dye-injected JO afferents from two different specimens are reconstructed and registered into the Honeybee Standard Brain (HSB). The soma of the DL-Int-1 neuron is located in the dorsal, most posterior region of the protocerebral lobe and posterior to the central body (transparent yellow, CB). DL-Int-1 has dense arborizations in the dorsal lobe and dorsal subesophageal ganglion (DL-dSEG) and sends a small branch into the medial posterior protocerebral lobe (medial PPL). (**C**) Changes in the vibration response pattern of a DL-Int-1 before and after olfactory stimuli to the contralateral antenna. (C-a) Vibration applied to the ipsilateral antenna causes an on-off phasic excitation (arrowheads). (C-b) Olfactory stimulation (orange odor) applied to the contralateral antenna causes a long-lasting excitation. (C-c) Vibration applied to the ipsilateral antenna after long-lasting excitation shown in (b) causes a tonic inhibition (arrow). (**D**) Difference in response pattern of a DL-Int-1 neuron depending on the spontaneous spike frequency. (upper panel) the on-off phasic excitation evoked by vibration. (lower panel) the tonic inhibition evoked by vibration during injecting the positive current (1 nA) into this neuron. AL, antennal lobe; SEG, subesophageal ganglion. Modified from Ai *et al.* [43].

DL-Int-1 is one of the local interneurons in the DL-dSEG. This neuron has dense arborizations in the DL-dSEG and small branches in the medial PPL (Figure 5A,B). The arborization in the DL-dSEG is composed not only of spiny terminals but also of blebby terminals, while those in the medial PPL are composed of only spiny terminals. The sensory afferents of JO run close to the DL-Int-1 in the DL-dSEG (Figure 5A,B).

The response patterns of DL-Int-1 to vibration and odor stimulation are quite interesting. DL-Int-1 has spontaneous activities and shows on-off phasic excitation to vibration stimuli (Figure 5C-a, and long-lasting excitatory response to olfactory stimuli (Figure 5C-b). When the vibration stimulation is applied again after the olfactory stimulation, the neuron shows a tonic inhibition (Figure 5C-c). During positive current injections into DL-Int-1, the neuron also shows tonic inhibition to the vibration stimulus (Figure 5D) [43]. These results suggest that DL-Int-1 changes between two response patterns to the vibration stimuli—on-off phasic excitation and tonic inhibition—depending on the membrane potential.

#### 3.4.2. DL-Int-2

**Figure 6.**
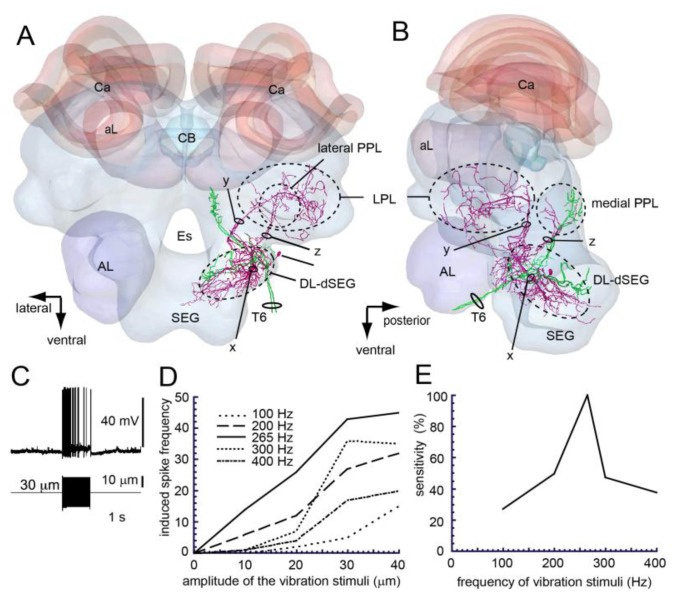
(**A** and **B**) Spatial relationship between a DL-Int-2 (magenta) and Johnston's organ (JO) afferents (green) (A, frontal view; B, lateral view). The soma is located in the posterolateral region of the dorsal lobe (DL). The neuron has three major ramifications (x, y, and z). The most strongly ramified arborizations (x) are arborizing in the DL and dorsal subesophageal ganglion (DL-dSEG) with numerous fine spines. A long process (y) terminates in the lateral protocerebral lobe (LPL) with fine presynaptic terminals. A small branch (z) emanates from the major DL branch and projects into the lateral portion of the posterior protocerebral lobe (lateral PPL) with fine presynaptic terminals. LPL, lateral protocerebral lobe. (**C**) DL-Int-2 showed tonic excitation in response to vibratory stimulation. (**D**) Relationship between the amplitude of vibratory stimulation of the antenna and the evoked spike numbers per second of DL-Int-2 neurons. Vibration with amplitudes of 10-40 μm was applied because the evoked spike frequency was saturated at c.a. 40 μm. The frequency of vibrations ranged from 100-400 Hz. The horizontal dotted line shows the response threshold of 15/s (spike frequency; 15 Hz) and the vertical dotted lines show the respective amplitudes (x) of vibrations for the different frequencies. (**E**) Sensitivity of DL-Int-2 to each applied vibratory frequency. The sensitivity was the reciprocal of each x, normalized to the reciprocal of x at 265 Hz (=100%). DL-Int-2s were more sensitive to 265 Hz vibrations applied to Johnston's organ than to vibrations of other frequencies. Modified from Ai *et al.* [43].

DL-Int-2 is one of the output neurons arborized in the primary auditory center. This neuron has arborizations in the DL-dSEG, PPL and lateral protocerebral lobe (LPL) (Figure 6A,B) [43]. This neuron shows phasic-tonic excitatory response to vibration stimuli (Figure 6C). The spike frequencies increase depending on the amplitude and the frequency of the vibration stimuli (Figure 6D). The most sensitive vibration frequency is 265 Hz, which is the frequency created during the waggle dance (Figure 6E) [43].

#### 3.4.3. PPL-D-1

**Figure 7.**
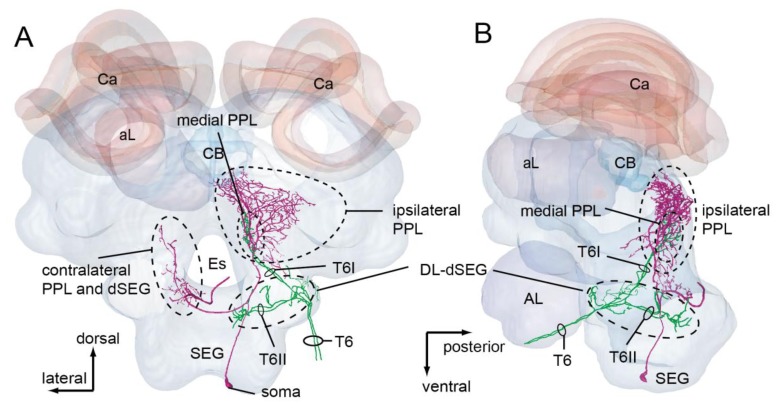
(**A** and **B**) Spatial relationship between a PPL-D-1 (magenta) and Johnston's organ (JO) afferents (green) (A, frontal view; B, lateral view). The soma of the PPL-D-1 is located in the ventral median region of the SEG. PPL-D-1 overlaps with the terminal branches of the JO in the medial PPL. The PPL-D-1 has dense and broad arborizations in ipsilateral PPL and sparse and fine arborizations with presynaptic terminals in the contralateral PPL and dorsal SEG. (**C**) On the first vibration stimulation, the PPL-D-1 does not respond to vibration applied to the antenna. The second vibration stimulus during olfactory stimulation (citral) applied to the contralateral antenna causes a long-lasting excitation. Modified from Ai *et al.* [43].

PPL-D-1 is one of the descending neurons arborized in the ipsilateral PPL. This neuron has arborizations in the ipsilateral PPL, contralateral PPL and dSEG (Figure 7A,B) [44]. This neuron did not respond to the 265 Hz vibration, but responded instead to vibrations with long-lasting excitation during olfactory stimulation to the contralateral antenna (Figure 7C) [45].

## 4. Neural Circuits of Vibration Processing

Neuroanatomical experiments showed that DL-Int-1 and DL-Int-2 have close appositions with JO afferents in dSEG (Figure 5 and 6). Moreover studies using a honeybee standard brain (HSB) [46] found that DL-Int-1 was close to JO afferents in the center of the DL, while DL-Int-2 was close to JO afferents in the anterior region of the DL [44]. DL-Int-1 shows on-off phasic excitation or tonic inhibition to vibration stimuli at 265 Hz, while DL-Int-2 shows tonic excitation to vibration at 265 Hz [43]. These morphological and physiological results suggest that these vibration-sensitive interneurons could be related to vibration processing in the primary center of the JO. In the olfactory primary center antennal lobe there are two types of interneurons: local interneurons and output neurons [47,48]. It is interesting that there are the same local interneurons (DL-Int-1) and output neurons (DL-Int-2) in the vibration primary center DL as in the olfactory primary center.

DL-Int-1 has two response patterns to vibration stimuli to the antenna [43]: on-off phasic excitation to vibration stimuli (on-off E) and tonic inhibition (tonic I; Figure 5C). HSB analysis revealed that the dendritic arborizations of the DL-Int-1 were in close proximity to the JO afferents in the DL (Figure 5A,B), and this on-off E was possibly evoked through the direct excitatory synapses from the JO afferents to the DL-Int-1.

Moreover, the tonic I was evoked by vibration stimuli, so that DL-Int-1 should receive inhibitory inputs from inhibitory interneurons. Since GABA-immunoreactive fibers exist in the DL-dSEG [49], it is suggested that DL-Int-1 receives synaptic inputs from these GABAergic neurons. Because two response patterns of the DL-Int-1 to the vibration stimuli were reversibly changed depending on the spontaneous spike firing rate [43], it is speculated that inputs from other sensory systems might modulate the response patterns of DL-Int-1 to vibration stimuli.

**Figure 8.**
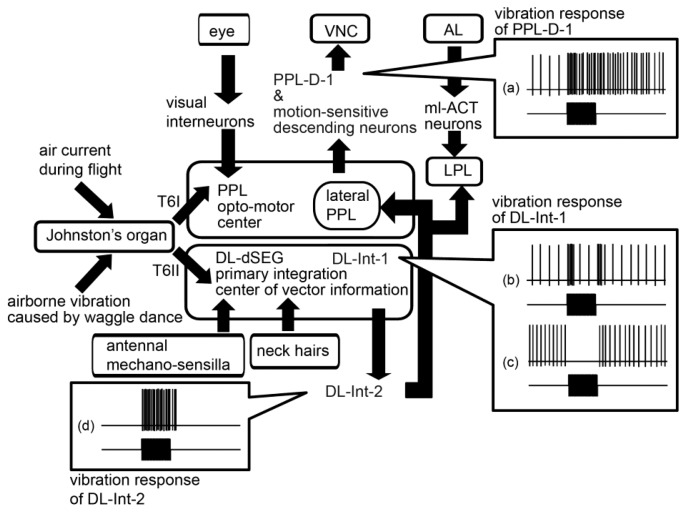
Scheme of processing pathways of mechanosensory signals detected by Johnston's organ (JO). The JO detects airborne vibrations during the waggle dance and also air-currents during flight. The JO afferents send this information to the PPL and DL-dSEG, through T6I and T6II, respectively. The PPL is thought to be an optomotor reflex center, which has axon terminals of visual interneurons and also has a dendritic arborization of motion-sensitive descending neurons. PPL-D-1 also has a dendritic arborization in the PPL and responds with a long-lasting excitation to vibration stimuli to the ipsilateral antenna during olfactory stimuli to the contralateral antenna (a). On the other hand, the DL-dSEG is thought to be a primary integration center of vector information encoded in the waggle dance, which has axon terminals of mechanosensilla on the antenna and also has those of neck hairs (gravity sense organ). DL-Int-1 is a kind of local interneuron in the primary centers and responds to vibrations with on- and off-phasic excitation (b). After olfactory stimulation to the contralateral antenna, the DL-Int-1 responds as a tonic inhibition to vibratory stimulation (c). DL-Int-2 is a kind of output neuron sending the axon to the LPL and lateral PPL and responds to vibrations with a tonic excitation (d). The LPL also has axon terminals of ml-ACT neurons, which is one of the olfactory interneurons originating from the AL. Modified from Ai and Itoh [45] and Ai and Hagio [42].

DL-Int-2 receives excitatory inputs by vibration stimuli to the antenna [43]. DL-Int-2 is most sensitive to vibrations at 265 Hz, which is the main frequency of airborne vibrations during the waggle dance, and the spike frequency increases depending on the amplitude of the vibration stimuli. HSB analysis revealed that the dendritic arborization of the DL-Int-2 is in close proximity to the JO afferents in the DL (Figure 6A,B), and the DL-Int-2 might also receive direct excitatory synaptic inputs from the JO afferents. Moreover, DL-Int-2 has axons with blebby terminals in the LPL and PPL. The LPL is one of the secondary centers of olfaction [50], while PPL is the secondary center of vision [51]. It is suggested that DL-Int-2 has a role in the transfer of information, encoded in vibrations detected by the JO, into the secondary centers of olfaction and vision (Figure 8).

PPL-D-1 extends the dendritic arborizations in the ipsilateral PPL (Figure 7A,B). However, there are no overlapping branches with those of DL-Int-1 and DL-Int-2. Conversely, JO afferents running in the T6I are in close proximity to some branches of the PPL-D-1 in the medial PPL, as revealed by using HSB analysis (Figure 7A,B). JO afferents possibly have synaptic contacts with JO afferents. PPL-D-1 was not responsive to vibration stimuli to the antenna, but began responding to vibration stimuli with long-lasting excitation during olfactory stimuli to the contralateral antenna (Figure 7C). The interneurons extending from the contralateral AL and terminating in the ipsilateral PPL might modulate the synaptic transmission from the JO to the PPL-D-1 (J. Rybak, personal communication).

## 5. Parallel Processing Pathways of Information Encoded in Vibrations

The pathways of the mechanosensory information detected by the JO, as found in our neuroanatomical and neurophysiological research, are shown in Figure 8. There are remarkable parallel pathways from the JO afferents to the vibration-sensitive interneurons in the primary center of JO. One is the pathway through the opto-motor reflex center PPL (PPL pathway). Previous studies have identified many motion-sensitive interneurons and descending neurons arborized in the PPL [51]. That the sensory afferents passing through T6I extend to the medial PPL suggests that the medial PPL is the integration center of the visual information detected by ocelli and compound eyes, and the mechanosensory information detected by the JO. The giant fiber neuron (GFN) in fruit fly has an arborization in a part of primary auditory center, called zone AB [52]. The GFN mediates the light-off escape response by relaying excitation from the eyes to the muscles of the thorax which are related with jumping and flying [53,54]. The medial PPL in honeybees has the similar neuropile of the zone AB in fruitfly.

The other is the pathway through the DL-dSEG, which is the terminal region of afferents of both contact-mechanosensing by antennae and gravity-sensing by NHs (DL-dSEG pathway). It has been suggested that mechanosensing is related to reception of vector information encoded in the waggle dance. Therefore, it is suggested that the DL-dSEG could be a region for integrating the vector information received by the JO and NHs during waggle dance communication. DL-Int-1 and -2, which have dendritic arborizations in the DL-dSEG, remarkably changed the spike frequencies at on and off timings of vibration stimuli. These results suggest that both DL interneurons have roles in monitoring the duration of vibrations (Figure 8B–D). The duration of airborne vibrations created in the waggle dance is one of the parameters denoting distance to the indicated flower [32]. These DL interneurons are possibly related to the neural circuits that decipher distance to the indicated flower by detecting the duration of vibrations. Thus the information detected by the JO of honeybees is transferred through parallel pathways (PPL pathway and DL-dSEG pathway) in the primary center. Such parallel pathways have been found not only in the auditory system of crickets [55,56], but also in the olfactory system of honeybees [50,57,58], of cockroach [59,60], of moth [61–64], of fly [65–68] and of ants [69,70]. These parallel processing might be a common characteristic in the olfactory and auditory systems of insects.

## 6. Conclusions

Airborne vibrations are used by silkmoths to regulate wingbeats, and by honeybees also to communicate vector information. In this final section we will summarize the characteristics of vibration processing.

1. On the sensory level: wing-margin bristles in silkmoth have roles in frequency filtering and direction filtering, and Johnston's organ of honeybees plays a role in sharpening of frequency tuning.
2. On the central nervous system (CNS) level: honeybees have parallel processing for the distinct behaviors of flight and communication.

These characteristics suggest that these insects have sensory systems equipped for executing quick adaptive responses to the environment. On the sensory level, the critical features of the signals were extracted depending on how the sensory signals are used for a specific behavior. Therefore, on the CNS level, the quick adaptive responses are made possible by combining the extracted sensory signals with the motor program or by integration with other sensory signals. The JO afferents in honeybees output to several centers in the CNS and different processing is executed at each center for flight or for deciphering vector information. Since the bristles along the wing margin are common among the lepidopterous species, the lepidpterous species have similar sensory system during the flight. And the fruitfly and mosquitoes have Johnston's organ for detecting airborne vibration and also have parallel processing system in the brain. The insect sensory system is considered to be a sophisticated system that requires minimum essential processing for very sensitive sensing, as well as quick and accurate responses. Insects have evolved such sensory-CNS systems under the limitations of a small body size and brain. After further studies, it will become clear what kinds of algorithms are used by the sensory-CNS system of insects in solving problems.
